# Association between 14 candidate genes, PM2.5, and affective disorders: a study of the Taiwan Biobank

**DOI:** 10.1186/s12889-023-16764-8

**Published:** 2023-11-27

**Authors:** Kai-Jie Ma, Yi-Ju Lin, Chiu-Shong Liu, Pei-Ying Tseng, Shi-Heng Wang, Chi-Yu Yao, Jong-Yi Wang

**Affiliations:** 1 Department of Public Health, China Medical University, Taichung, Taiwan; 2 Department of Administration, China Medical University Hospital, Taichung, Taiwan; 3 Department of Family Medicine, China Medical University Hospital, Taichung, Taiwan; 4 Department of Medicine, China Medical University, Taichung, Taiwan; 5 Department of Medical, Lee’s General Hospital, Yuanli Town, Miaoli, Taiwan; 6 Interdisciplinary Freshmen Program of Public Health, China Medical University, Taichung, Taiwan; 7 Attending physician Department of psychiatry, An-nan hospital, Tainan, Taiwan; 8 Department of Health Services Administration, China Medical University, Taichung, Taiwan

**Keywords:** Affective psychosis, Gene, Single nucleotide polymorphism (SNP), Genome-wide, Environmental risks

## Abstract

**Background:**

Most studies have focused on the risk factors, treatment, and care of affective psychosis, and several have reported a relationship between ambient air quality and this psychosis. Although an association has been reported between psychosis and genes, studies mainly explored the associations between one type of psychosis and one gene; few have identified genes related to affective psychosis. This study investigates the genetic and environmental factors of affective psychosis.

**Methods:**

In this retrospective longitudinal study, 27 604 participants aged 30–70 were selected from Taiwan Biobank. The participants’ propensity scores were calculated based on their demographic information, and propensity score matching was performed to divide the participants into an experimental (i.e., affective psychosis) and control group at a 1:5 ratio. Plink was used to analyze the major and minor types of gene expression related to affective psychosis, and PM_2.5_ exposure was incorporated into the analyses.

**Results:**

According to the generalized estimating equation analysis results, 8 single nucleotide polymorphisms (SNPs) belonging to the ANK3, BDNF, CACNA1C, and GRID1 genotypes were significantly correlated with depressive disorder (*P* < .001), with the majority belonging to the ANK3 and CACNA1C. A total of 5 SNPs belonging to the CACNA1C, GRID1, and SIRT1 genotypes were significantly correlated with bipolar disorder (*P* < .001), with the majority belonging to the CACNA1C. No significant correlation was identified between ambient air pollution and affective psychosis.

**Conclusions:**

CACNA1C and GRID1 are common SNP genotypes for depressive disorder and bipolar disorder and should be considered associated with affective psychosis.

**Supplementary Information:**

The online version contains supplementary material available at 10.1186/s12889-023-16764-8.

## Introduction

The World Health Organization declared affective psychosis to be a disease of the 21st century. Affective psychosis can be divided into depressive disorder and bipolar disorder. Approximately 280 million people worldwide have been given a diagnosis of depressive disorder 1, and 45 million have been given a diagnosis of bipolar disorder 2. Medical data from the past decade have revealed a considerable increase in the number of patients in Taiwan who are given diagnoses of affective psychosis. However, affective psychosis is stigmatized worldwide 3; many individuals refuse to acknowledge having affective psychosis, leading to numerous potentially untreated cases.

Genetics and mental health research has revealed that 5 types of common psychosis, namely autism, attention deficit hyperactivity disorder, bipolar disorder, schizophrenia, and depressive disorder, share a common specific genetic variant 4. Studies have reported that genetic defects can influence brain growth, causing symptoms of psychosis. Psychosis is also associated with other genetic and environmental factors; Specific genes have been linked to depressive disorder severity 5. In 2020, Amare conducted a meta-analysis of genome-wide association studies and verified that depressive disorder and bipolar disorder are genetically inherited 6. Bipolar disorder (BD) involves drastic mood swings alternating between manic and depressive episodes, which can severely impair the functional status, quality of life, and interpersonal relationships of patients and their families. The lifetime and 12-month prevalence of bipolar disorder is 2.4 and 1.5%, respectively 7; its heritability is 70–90%. Studies have verified 30 loci as been associated with bipolar disorder 8, although these loci exhibit small effect sizes. Studies have also reported an association between genetic variation and environmental factors 9. In addition to genetics and the environment, past literature has indicated that factors such as gender, age, income, smoking, and alcohol consumption are associated with depression. Studies have shown that females are more susceptible to depression compared to males 10, 11, and there is a correlation between age and the incidence of depression 12. Income levels are also believed to influence the risk of depression 13, 14, and behaviors such as smoking 15 and alcohol consumption 16 are considered to be related to the onset of depression. Furthermore, previous research has highlighted other factors that are relevant to the occurrence of both depression and BD. Firstly, exercise habits have been proven to have a positive impact on preventing depression. Regular physical activity can improve psychological well-being, reduce stress and anxiety, and contribute to lowering the risk of depression 17, 18. Secondly, there is a correlation between education level, place of residence, and BD. Education level may play a role in influencing the risk of developing BD 19, and the social environment and cultural background of the place of residence may also be related to the incidence of BD 20, 21. Additionally, marital status and betel nut consumption have also been associated with mental disorders. Marital status may affect psychological health and be linked to the incidence rates of mental disorder 22. Betel nut consumption has been found to be possibly associated with specific mental health risks 23.

Among the five common mental disorders, depression and BD have notably high prevalence rates. Globally, it is estimated that approximately 5% of adults suffer from depression 24, and 4.4% of U.S. adults experience bipolar disorder at some point in their lives 25. The prevalence of both depression and BD continues to rise in Taiwan as well. Additionally, BD is characterized by episodes of mania and depression, and during the depressive phase, patients exhibit symptoms similar to those of major depression. Therefore, we aim to investigate both depression and BD simultaneously in this study to gain a comprehensive understanding of these two mental disorders. In this study, the genetic factors leading to affective disorder in addition to the factors leading to affective psychosis, such as environmental factors, were investigated. Elucidating the relationship between genes and affective psychosis will enable individuals to understand which are high-risk genes for affective psychosis prior to pregnancy, reducing the risk of affective disorder in newborn children due to genetic transfer. It will also enable people with high risks of affective psychosis to identify this risk through genetic testing, which will enable them to gain preventive medical knowledge before the onset of the disorder and receive appropriate interventions, such as psychotherapy and health management and thereby prevent the negative effects of the disorder.

## Methods

### Study population

This study focused on individuals who completed Taiwan Biobank whole-genome database questionnaires between 2012 and 2016. Those with and without affective psychosis were categorized into experimental and control groups, respectively. Data on the average PM_2.5_ concentration in the preceding year, as reported in the environmental resource databases of the Environmental Protection Administration, from 2011 to 2016 were used as ambient air quality data. Thirteen variables were applied for propensity score matching (PSM), namely sex, age, income, education level, marital status, comorbidity, health behaviors (smoking, drinking, and betel nut consumption and exercise habits), ambient air quality, regional characteristics, and place of residence.

Candidate genes.

Our research source is the Taiwan Biobank, which is a model database developed in accordance with the Human Biobank Management Act. The Taiwan Biobank was designed as a multicenter cooperative alliance for data collection and is capable of continuous data collection. This domestic Taiwanese human biological database was developed by tracking and updating people’s lifestyle, health status, clinical treatment, medication information, disease progression, and biological sample data. All patients participating in the Taiwan Biobank sign an informed consent and participation consent form after receiving a complete description. Additionally, the Taiwan Biobank has been certified by the ISO/IEC 27001:2013 information security management system to ensure the protection of privacy and personal information. Our study was also approved by the Institutional Review Board (CRREC-108-006), and we followed relevant guidelines and regulations. For this study, samples collected from 2012 to 2016 from a total of 27,604 individuals aged 30 to 70 years and without foreign ancestries were employed. We conducted genotyping on all samples using custom-made Affymetrix Axiom genome-wide array plates (Affymetrix Inc., Santa Clara, CA, USA), referred to as TWB arrays. These arrays encompass SNPs for all candidate genes. We identified 14 candidate gene research that has shown possible associations with affective psychosis phenotypes from the peer-reviewed biomedical literature indexed in PubMed (Table S1). During the genotyping process, single nucleotide polymorphisms (SNPs) that did not meet quality control criteria were excluded. SNPs with a minor allele frequency (MAF) < 0.05, a call rate < 95%, and those that deviated from the Hardy-Weinberg equilibrium (HWE) with a p-value < 1.0 × 10^− 3^ were removed from the analysis. Healthy participant questionnaires were referenced to obtain data on the individuals’ health behaviors and diets.

### Ambient air quality

Air pollution exposure assessment and concentration data from 2011 to 2016 were collected from the environmental resource databases of the Environmental Protection Administration and were used as the source of ambient air quality data.

### Statistical analysis

Plink was applied to analyze and manage the genome-wide association data. The data were converted to a single nucleotide polymorphism (SNP) data format, and SNP quality was controlled in the SNP statistics. Statistical Analysis Software 9.4 was employed for statistical analysis. Descriptive statistics were employed to present the data distribution. To prevent overly high correlations between variables, which could hinder thorough estimation of the regression model parameters, collinearity diagnostics were performed followed by an inferential statistical analysis. To improve estimation accuracy, PSM was performed in experimental and control groups, with a ratio of 1:5 for sex, age, income, marital status, education level, occupation, health behaviors, comorbidities, regional characteristics, place of residence, and ambient air pollution concentrations. A Student’s *t* test, Χ 2 test, and binary logistic regression analysis was performed to analyze potential correlations between affective psychosis and the dependent variables. The generalized estimation equation was adopted to analyze potential correlations between genetic polymorphism, ambient air pollution exposure, and psychosis diagnosis.

## Results

### Participant characteristics

The whole-genome data from Taiwan Biobank for 27 604 individuals were determined to be suitable for this study. 49.9% and 50.1% of the participants were men and women, respectively; all participants were aged ≥ 30 years. According to the PM_2.5_ exposure monitoring data of the Environmental Protection Administration, ambient air quality was divided into 3 levels; 68.1% of the participants were exposed to the PM_2.5_ concentration of 15.5 to 35.4 µg/m^3^ (Table 1).

**Table 1:** Person characteristics of subjects (N = 27,604)

Variables	N		%
**Gender**			
Male	13,766		49.9%
Female	13,838		50.1%
**PM**_**2.5**_ **expose**			
0-15.4 µg/m^3^	300		1.1%
15.5–35.4 µg/m^3^	18,791		68.1%
35.5–54.4 µg/m^3^	8,513		30.8%
**Gene**	**SNP**		
ANK3		2,920	
BACE1		79	
BDNF		204	
CACNA1C		2,368	
COMT		147	
CRHR1		225	
GRID1		3,272	
IGF1		183	
ODZ4		2,976	
PER3		291	
SHANK3		97	
SIRT1		127	
TLR4		32	
TPH1		51	

### Gene and SNPs

We categorized each SNP into major type and minor type based on their frequency distribution in the sample. Taking the SNP rs1002442 within the ANK gene as an example, its SNP frequencies are as follows: GG accounts for 0.6%, GT account for 13.9%, and TT accounts for 85.3%. Therefore, we classify the TT genotype as the major type, while the GG and GT genotypes are considered the minor types in terms of gene expression (Table 1).

### Depressive disorder

The Χ 2 and *t* tests revealed strong, significant correlations between several variables and depressive disorder. However, because the variables differed considerably in their sample distributions, each variable underwent PSM to eliminate the uneven sample distribution. The results revealed that the participants who were men (OR = 2.04), had low-income jobs (OR = 0.62–0.85 for men with an income over NT$20 000), were single (OR = 0.60 for men who were married), smoked (OR = 1.69), had severe comorbidity (OR = 1.83–5.34), lived in regions with low urbanization (OR = 1.82–1.98), and lived in central Taiwan (OR = 1.25) had the highest risks of depressive disorder(Table 2).

**Table 2:** Association between Participant Characteristics and Depressive Disorder

	Depressive disorder group		OR	95% CI
	Before PSM	After PSM		
**PM**_**2.5**_ **exposure**	0.565	0.935	0.99	0.97–1.01
**Sex**	< 0.001	0.178		
F			-	-
M			2.04**	1.71–2.44
**Income**	< 0.001	0.717		
<NT$20 000			-	-
NT$20 000–30 000			0.85	0.62–1.16
NT$30 000–40 000			0.64*	0.45–0.91
NT$40 000–50 000			0.62*	0.42–0.92
>NT$50 000			0.68*	0.50–0.90
Missing			0.66*	0.50–0.88
**Marital status**	< 0.001	0.530		
Single			-	-
Married			0.60**	0.49–0.74
**Education level**	< 0.001	0.977		
Elementary and below (noncompletion included)			-	-
Junior high and above (noncompletion included)			1.47*	1.03–2.11
**Comorbidity**	< 0.001	0.543		
0			-	-
1			1.83**	1.57–2.14
2			2.81**	2.11–3.75
3 or more			5.34**	2.99–9.55
**Regional characteristics**	0.001	0.980		
Metropolis			-	-
Medium or emerging city			1.46	0.96–2.21
Town			1.82*	1.20–2.75
Remote area			1.98*	1.29–3.03
**Place of residence**	0.187	0.927		
Northern Taiwan			-	-
Central Taiwan			1.25*	1.03–1.52
**Smoking**	0.013	0.099	1.69**	1.42–2.01

* P-value < 0.05

** P-value < 0.001

No collinearity was discovered between any variables (VIF < 100)

Logistic regression: *P* > .05 for age; marital status (including divorced, separated, and widowed); education level (including high school, university, and graduate school); place of residence (including southern Taiwan, eastern Taiwan, and outlying islands); drinking, betel nut consumption, dietary habits and exercise habits (Table S2)

### Bipolar disorder

The variables underwent PSM with respect to their correlations with bipolar disorder to eliminate uneven sample distribution. No collinearity was identified between any of the variables. Regression analysis revealed that the participants who were men (OR = 1.94), had low-income jobs (OR = 0.55), were single (OR = 0.39 for men who were married), had low education levels (OR = 0.52 for high school; OR = 0.28 for graduate school), had severe comorbidity (OR = 1.99–3.85), and who smoked (OR = 2.28) had the highest risks of bipolar disorder(Table 3).

**Table 3:** Association between Participant Characteristics and Bipolar Disorder

	Bipolar disorder group		OR	95% CI
	Before PSM	After PSM		
**PM**_**2.5**_ **exposure**	0.340	0.336	1.00	0.96–1.04
**Sex**	0.015	0.41		
F			-	-
M			1.94*	1.32–2.84
**Income**	0.093	0.671		
<NT$20 000			-	-
NT$20 000–50 000			0.55*	0.31–0.96
**Marital status**	< 0.001	0.469		
Single			-	-
Married			0.39**	0.26–0.59
**Education level**	0.004	0.990		
Elementary and below (noncompletion included)			-	-
Junior high (noncompletion included)			0.61	0.30–1.22
Senior high or vocational school (noncompletion included)			0.52*	0.29–0.92
College or university (noncompletion included)			0.61	0.34–1.10
Graduate school (noncompletion included)			0.28*	0.11–0.70
**Comorbidity**	< 0.001	0.778		
0			-	-
1			1.99**	1.42–2.79
2 or more			3.85**	2.30–6.43
**Smoking**	0.001	0.784	2.28**	1.58–3.29

* P-value < 0.05

** P-value < 0.001

No collinearity was discovered between any variables (VIF < 100)

Logistic regression: P > .05 for age; income (including > NT$50 000 and missing); marital status (including divorced, separated, and widowed); regional characteristics; place of residence; drinking, betel nut consumption, and exercise habits

### Generalized Estimation Equation

The analysis results revealed that the minor gene expression types of rs142101917 were expressed in 8.18% and 5.29% of the depressive disorder and control groups, respectively. Relative to the major, the minor gene expression types of rs142101917 exhibited an OR of 1.60 for risk of depressive disorder. Relative to the major, the minor gene expression types of rs191244436, rs541490076, rs193069165, rs116938681, rs188470158, rs565998563, and rs558147168 exhibited ORs of 3.11, 2.60, 2.18, 2.60, 2.68, 1.84, and 2.92 for risk of depressive disorder, respectively. Furthermore, rs191244436, rs541490076, and rs193069165 belonged to the ANK3 genotype; rs193069165 belonged to the BDNF genotype; rs116938681, rs188470158, and rs565998563 belonged to the CACNA1C genotype; and rs558147168 belonged to the GRID genotype. A total of 4 genotypes with high risks of depressive disorder were identified (Table 4).

**Table 4:** Association between Genes and Affective Psychosis using Generalized Estimating Equations

Gene	SNP	Affective psychosis		GEE OR	95%CI	*p value*
		Y	N			
**Depressive disorder**						
ANK3	rs142101917	73(8.18)	236(5.29)	1.60	1.22–2.09	6.93*10^− 4^
ANK3	rs191244436	19(2.13)	31(0.70)	3.11	1.76–5.49	9.22*10^− 5^
ANK3	rs541490076	20(2.24)	39(0.87)	2.60	1.52–4.46	5.18*10^− 4^
BDNF	rs193069165	27(3.03)	63(1.41)	2.18	1.38–3.44	8.51*10^− 4^
CACNA1C	rs116938681	21(2.35)	41(0.92)	2.60	1.52–4.44	4.80*10^− 4^
CACNA1C	rs188470158	18(2.02)	34(0.76)	2.68	1.50–4.79	8.70*10^− 4^
CACNA1C	rs565998563	63(7.06)	177(3.97)	1.84	1.37–2.46	4.46*10^− 5^
GRID1	rs558147168	23(2.58)	40(0.90)	2.92	1.75–4.88	3.88*10^− 5^
**Bipolar disorder**						
CACNA1C	rs541349080	11(6.15)	15(1.68)	3.84	1.82–8.12	4.26*10^− 4^
CACNA1C	rs7295590	115(64.25)	456(50.95)	1.73	1.25–2.39	9.31*10^− 4^
CACNA1C	rs7979389	115(64.25)	456(50.95)	1.73	1.25–2.39	9.31*10^− 4^
GRID1	rs145322445	15(8.38)	30(3.35)	2.64	1.49–4.67	8.63*10^− 4^
SIRT1	rs35648458	9(5.03)	10(1.12)	4.69	2.05–10.71	2.53*10^− 4^

The ORs of rs541349080, rs7295590, rs7979389, rs145322445, and rs35648458 for risk of bipolar disorder were 3.84, 1.73, 1.73, 2.64, and 4.69, respectively. Accordingly, the CACNA1C, GRID1, and SIRT1 genotypes were identified as carrying the highest risks of bipolar disorder.

The general estimation equation analysis results revealed no statistical significance between ambient air pollution severity in the preceding year and the risk of affective psychosis. Therefore, PM_2.5_ exposure was not correlated with affective psychosis (Table 5).

**Table 5:** Association between Air Pollution Exposure Assessment and Affective Psychosis using Generalized Estimating Equations

PM_2.5_	Depressive disorder				Bipolar disorder		
	*n* = 892	GEE OR	95%CI		*n* = 179	GEE OR	95%CI
**PM**_**2.5**_ ** µg/m** ^**3**^	30.6 ± 7.4	0.996	0.987–1.004		30.7 ± 7.8	0.99	0.98–1.01
**PM**_**2.5**_ **group**							
0–15.4	13	-	-		4	-	-
15.5–35.4	603	0.902	0.532–1.531		120	0.81	0.31–2.10
35.5–54.4	276	0.916	0.533–1.572		55	0.63	0.25–1.63

## Discussion

### Gene

Eight SNPs were identified as associated with depressive disorder and belonged to the ANK3, BDNF, CACNA1C, and GRID1 genotypes, with the majority belonging to the ANK3 and CACNA1C genotypes. Five SNPs were identified as associated with bipolar disorder and belonged to the CACNA1C, GRID1, and SIRT1 genotypes, with the majority belonging to the CACNA1C genotype. Accordingly, CACNA1C and GRID1 were identified as common genes associated with both bipolar disorder and depressive disorder, suggesting that different types of psychosis not only have their own associated genes but also have common genes. This is consistent with the findings of other research, which indicate that the genetic basis of psychopathological dimensions is shared between different types of affective psychosis 5.

The results of this study indicate that affective psychosis can be identified through different genes in each ethnicity. One study including Pakistani people indicated that rs1006737, which belongs to the CACNA1C genotype, was associated with BD 26. By comparison, a study including Japanese people reported that ODZ4 was associated with bipolar disorder 27. Another study including 12 000 Han Chinese women revealed 2 genes (SIRT1 and LHPP) to be significantly associated with severe depressive disorder. An analysis of the gene subtypes indicated that the gene most significantly associated with severe depressive disorder was SIRT1^28^. A study including British people revealed that SLC6A5, BDNF, COMT, and HTR2A were not candidate genes for severe depressive disorder 29. These findings differed from those of this study, verifying the homogeneity of the genes associated with affective psychosis in different ethnicities. Because this study solely focused on Taiwanese people, its results were limited.

In a recent study involving joint analysis of population-based and family-based research, it was observed that 5-HTTLPR has a marginal but detectable impact on bipolar disorder (BD). 5-HTTLPR is considered a useful predictor of antidepressant response, but it cannot predict responses to antidepressant or antimanic treatments 30. Another investigation exploring susceptibility variations related to suicide attempts in patients with major depressive disorder identified TPH2 as the top susceptibility gene in gene-based analyses. It is a robust biological candidate given its strong biological relevance in the serotonin system 31. In future research investigating affective psychosis, 5-HTTLPR and TPH2 can be included as a more promising candidate gene.

### Ambient air quality

Many studies have reported ambient air quality to be associated with affective psychosis. A 2020 study including Chinese people indicated that an increase in PM_2.5_ concentrations can lead to reduced social activity, which can enhance the severity of depressive disorder 32. A meta-analysis reported that long-term PM_2.5_ exposure increased anxiety, inhibiting psychological health and increasing the risk of suicide 33. However, the results of this study were more consistent with the findings of Fan 34, who reported that PM_2.5_ exposure was associated with neither bipolar disorder nor depressive disorder.

### Other person characteristics

The results of this study indicated a significant correlation between affective psychosis and demographic factors, with participants who were men, had low-income jobs, were single, had education levels no higher than junior high school, had severe comorbidity, and lived in regions with low urbanization or in central Taiwan being at the highest risk of depressive disorder. In addition, participants who were men, had low-income jobs, were single, had low education levels, and had severe comorbidity were at the highest risk of bipolar disorder. In 1981, Blum proposed 4 social determinants of health: environment, heredity, lifestyle, and health 35. In this study, an association was identified between health factors and risk of affective psychosis. The health behavior most associated with depressive disorder and bipolar disorder was smoking; depressive disorder was 1.69 times as common in participants who smoked compared with in those who did not, and bipolar disorder was 2.28 times as common in participants who smoked compared with in those who did not. Accordingly, smoking was identified as a common health risk factor for affective psychosis. This was consistent with the findings of other studies, which indicated that smoking increases the risk and symptoms of affective psychosis 36–38. Our research found that males are more susceptible to affective disorders, which differs from past research results. In the past, it was commonly believed that females were more prone to affective disorders 10, 11, making this new discovery particularly intriguing. Additionally, we also observed that individuals engaged in low-income jobs 39, living single 40, having a lower educational level (junior high school or below) 41, and those with severe comorbidities are more likely to suffer from depression 42, aligning with previous research findings. Furthermore, areas with low urbanization or in central Taiwan are more prone to experiencing depressive disorders. This could be attributed to the fact that central Taiwan is a relatively young and less urbanized region currently undergoing a developmental phase. Moreover, according to government statistical data 43, the number of incoming residents in central Taiwan has been consistently increasing in recent years, providing further evidence of the region’s developmental stage. Past research pointed out that populations in developing areas are more susceptible to depression 44.

## Conclusion

Among the 5 genotypes associated with affective psychosis, CACNA1C and GRID1 were identified as common genes for bipolar disorder and depressive disorder, and CACNA1 was identified as the genotype most highly associated with affective psychosis.

Eight SNPs were associated with depressive disorder, namely rs142101917, rs191244436, and rs541490076 of the ANK3 genotype; rs193069165 of the BDNF genotype; rs116938681, rs188470158, and rs565998563 of the CACNA1C genotype; and rs558147168 of the GRID1 genotype. Notably, most of these SNPs belong to the ANK3 and CACNA1C genotypes. Five SNPs were associated with bipolar disorder, namely rs541349080, rs7295590, and rs7979389 of the CACNA1C genotype; rs145322445 of the GRID1 genotype; and rs35648458 of the SIRT1 genotype. Most of these SNPs belonged to the CACNA1C genotype. Accordingly, CACNA1C and GRID1 were identified as common genotypes for bipolar disorder and depressive disorder.

### Suggestions

1. Many factors influence affective psychosis. Several genome-wide association studies have been performed to identify the SNPs related to affective psychosis. However, future studies should explore the interactions between genetic, variation, and environmental factors, and genome-wide association studies should be conducted to identify the genes associated with affective psychosis.
2. Education level is a common factor affecting depressive disorder and bipolar disorder. Future studies should further investigate the influence of education levels in Taiwan on people’s risk of affective psychosis to thereby provide a reference for governmental and educational agencies in planning curricula and education policies.
3. Sex is also a common factor affecting depressive disorder and bipolar disorder. Studies have reported that female participants are particularly vulnerable to affective psychosis; however, the statistical data from Taiwan Biobank indicated that male participants had higher rates of affective psychosis than female participants did. This finding should be incorporated as a key topic in future studies to achieve a more comprehensive understanding of the matter.

### Limitations

1. This study employed a questionnaire to collect data. Self-report questionnaires may incur statement biases.
2. People from different countries differ in their affective psychosis-related gene and lifestyle factors (e.g., diet, living habits, and healthy behaviors). Therefore, the results of this study are only applicable to the Taiwanese populations.
3. The “income” and “education level” factors from the model of social determinants of health cannot be extrapolated as generalized environmental factors.
4. The urbanization data employed in this study were drawn from the findings of other studies. Because of changes in the sociodemographic structure of Taiwan, the data may not accurately reflect current urbanization levels in Taiwan.
5. Regarding the selection of ambient air pollution monitoring stations, if a participant lived in a location without a monitoring station, the participant’s PM_2.5_ exposure for the preceding year could only be assessed according to corresponding county-level or city-level measurement values.

### Electronic supplementary material

Below is the link to the electronic supplementary material.

Supplementary Material 1

## Data Availability

The data that support the findings of this study are available from the Taiwan Biobank, but restrictions apply to the availability of these data, which were used under license for the current study, and so are not publicly available. Data are however available from the authors (Jong-Yi Wang, E-mail: Jong-Yi Wang) upon reasonable request and with permission of Taiwan Biobank.
