# Supplementary material for: Association between 14 candidate genes, PM2.5, and affective disorders: a study of the Taiwan Biobank
Source: BMC Public Health. 2023 Nov 27;23:2346. doi: 10.1186/s12889-023-16764-8 (PMC10683147; doi:10.1186/s12889-023-16764-8)
Supplement: Supplementary file 1 — Supplementary Material 1 [file 12889_2023_16764_MOESM1_ESM.docx]

| Table S1  Genes type associated with affective psychosis | | |
| --- | --- | --- |
| **Affective disorders** | **Gene** | **References** |
| Depressive disorder | CACNA1C | Green et al., 2010^1^ |
|  | CSF2RB | Chen P et al., 2011^2^ |
|  | SIRT1 | Cai et al., 2015^3^ |
|  | LHPP | Cai et al., 2015^3^ |
|  | SYNE1 | Rathje M et al., 2021^4^ |
|  | ANK3 | Khalid et al., 2018^5^ |
|  | BDNF | Youssef et al., 2018^6^ |
|  | TPH1 | Wigner et al., 2018^7^ |
|  | TPH2 | Wigner et al., 2018^7^ |
| Bipolar disorder | AVPR1B | L.Rodziewicz et al., 2013^8^ |
|  | CACNB2 | Chen J et al., 2014^9^ |
|  | CRHR1 | Bigdeli TB et al., 2021^10^ |
|  | CACNA1C | Khalid et al., 2018^5^ |
|  | ODZ4 | Orrù G et al., 2018^11^ |

| Table S2 | | | | |
| --- | --- | --- | --- | --- |
| Associated between participant characteristics and depressive disorder | | | | |
|  | Depressive disorder group | | OR | 95% CI |
|  | Before PSM | After PSM |  |  |
| **PM_2.5_ exposure** | 0.565 | 0.935 | 0.99 | 0.97–1.01 |
| **Sex** | <0.001 | 0.178 |  |  |
| F |  |  | - | - |
| M |  |  | 2.04** | 1.71–2.44 |
| **Income** | <0.001 | 0.717 |  |  |
| <NT$20 000 |  |  | - | - |
| NT$20 000-30 000 |  |  | 0.85 | 0.62–1.16 |
| NT$30 000-40 000 |  |  | 0.64* | 0.45–0.91 |
| NT$40 000-50 000 |  |  | 0.62* | 0.42–0.92 |
| >NT$50 000 |  |  | 0.68* | 0.50–0.90 |
| Missing |  |  | 0.66* | 0.50–0.88 |
| **Marital status** | <0.001 | 0.53 |  |  |
| Single |  |  | - | - |
| Married |  |  | 0.60** | 0.49–0.74 |
| Divorced or Separated |  |  | 1.25 | 0.97–1.62 |
| Widowed |  |  | 0.82 | 0.57–1.17 |
| **Education level** | <0.001 | 0.977 |  |  |
| Elementary and below (noncompletion included) |  |  | - | - |
| Junior high and above (noncompletion included) |  |  | 1.47* | 1.03–2.11 |
| High school |  |  | 1.28 | 0.92–1.76 |
| University |  |  | 1.07 | 0.77–1.50 |
| Graduate school |  |  | 1.09 | 0.73–1.64 |
| **Comorbidity** | <0.001 | 0.543 |  |  |
| 0 |  |  | - | - |
| 1 |  |  | 1.83** | 1.57–2.14 |
| 2 |  |  | 2.81** | 2.11–3.75 |
| 3 or more |  |  | 5.34** | 2.99–9.55 |
| **Regional characteristics** | 0.001 | 0.98 |  |  |
| Metropolis |  |  | - | - |
| Medium or emerging city |  |  | 1.46 | 0.96–2.21 |
| Town |  |  | 1.82* | 1.20–2.75 |
| Remote area |  |  | 1.98* | 1.29–3.03 |
| **Place of residence** | 0.187 | 0.927 |  |  |
| Northern Taiwan |  |  | - | - |
| Central Taiwan |  |  | 1.25* | 1.03–1.52 |
| Southern Taiwan |  |  | 0.99 | 0.74–1.32 |
| Eastern Taiwan |  |  | 1.62 | 0.99–2.64 |
| Outlying Islands |  |  | 0.71 | 0.22–2.26 |
| **Drinking** | 0.752 | 0.298 | 1.04 | 0.78–1.38 |
| **Smoking** | 0.013 | 0.099 | 1.69** | 1.42–2.01 |
| **Betel nut consumption** | 0.391 | 0.637 | 0.85 | 0.63–1.15 |
| **Exercise habits** | 0.498 | 0.401 | 1.06 | 0.92–1.23 |
| **Dietary habits** | 0.039 | 0.388 |  |  |
| Carnivorous |  |  | - | - |
| Vegetarian |  |  | 1.49 | 0.99–2.23 |
| * P-value<0.05, ** P-value<0.001 | | | | |

**References**

1 Green, E. K. *et al.* The bipolar disorder risk allele at CACNA1C also confers risk of recurrent major depression and of schizophrenia. *Molecular psychiatry* 15, 1016-1022 (2010).

2 Chen, P. *et al.* Common SNPs in CSF2RB are associated with major depression and schizophrenia in the Chinese Han population. *The World Journal of Biological Psychiatry* 12, 233-238 (2011).

3 Cai, N. *et al.* Sparse whole-genome sequencing identifies two loci for major depressive disorder. *Nature* 523, 588-591 (2015).

4 Rathje, M. *et al.* Genetic variants in the bipolar disorder risk locus SYNE1 that affect CPG2 expression and protein function. *Molecular psychiatry* 26, 508-523 (2021).

5 Khalid, M. *et al.* Association of CACNA1C with bipolar disorder among the Pakistani population. *Gene* 664, 119-126 (2018).

6 Youssef, M. M. *et al.* Association of BDNF Val66Met polymorphism and brain BDNF levels with major depression and suicide. *International Journal of Neuropsychopharmacology* 21, 528-538 (2018).

7 Wigner, P. *et al.* Association between single nucleotide polymorphisms of TPH1 and TPH2 genes, and depressive disorders. *Journal of cellular and Molecular Medicine* 22, 1778-1791 (2018).

8 Leszczyńska-Rodziewicz, A., Maciukiewicz, M., Szczepankiewicz, A., Pogłodziński, A. & Hauser, J. Association between OPCRIT dimensions and polymorphisms of HPA axis genes in bipolar disorder. *Journal of affective disorders* 151, 744-747 (2013).

9 Chen, J. *et al.* CACNB2 rs11013860 polymorphism correlates of prefrontal cortex thickness in bipolar patients with first-episode mania. *Journal of Affective Disorders* 268, 82-87 (2020).

10 Bigdeli, T. B. *et al.* Genome-wide association studies of schizophrenia and bipolar disorder in a diverse cohort of US veterans. *Schizophrenia bulletin* 47, 517-529 (2021).

11 Orrù, G. & Carta, M. G. Genetic variants involved in bipolar disorder, a rough road ahead. *Clinical practice and epidemiology in mental health: CP & EMH* 14, 37 (2018).
